# Reconstruction of Maxillary Defects Using Virtual Surgical Planning and Additive Manufacturing Technology: A Tertiary Care Centre Experience

**DOI:** 10.1007/s12663-023-02005-3

**Published:** 2023-09-15

**Authors:** Adarsh Kudva, G. Srikanth, Anupam Singh, A. Chitra, Ramya K. Suryanarayan, Mugdha Francis

**Affiliations:** Department of Oral and Maxillofacial Surgery, Manipal College of Dental Sciences, Manipal Academy of Higher Education (MAHE), Manipal, India

**Keywords:** Maxillary reconstruction, Virtual surgical planning, 3D printing technology, Additive manufacturing, Patient-specific implants

## Abstract

**Introduction:**

Maxillary reconstruction is often a challenging task for the surgeons because of the complex anatomy. However, with the advances in virtual surgical planning (VSP) and 3D printing technology there is a new avenue for the surgeons which offers a suitable alternative to conventional flap-based reconstructions.

**Patients and Methods:**

In this article, we have described 4 case scenarios which were managed with the help of VSP and additive manufacturing technology for complex maxillary reconstruction procedures. Use of the technologies aided the clinician in achieving optimal outcomes with regards to form, function and esthetics.

**Discussion:**

Virtual surgical planning (VSP) has gained a lot of impetus in past 1 decade. These aides the surgeon in determining the extent of disease and also carry out the treatment planning. In addition to VSP, the concept of additive manufacturing provides a viable alternative to the conventional reconstruction modalities for maxillary defect rehabilitation. Increased accuracy, rehabilitation of normal anatomical configuration, appropriate dental rehabilitation, decreased intra-operative time and post-operative complications are some of the advantages. In addition, patient-specific implants eliminate the need for a separate donor site. Apart from the treatment of pathologies, they also can be used for reconstruction of post-traumatic defect, where endosteal implant placement is not possible.

**Conclusion:**

These modalities show promising results for reconstruction of complex maxillary defects.

## Introduction

Maxillary defect can occur due to an extensive traumatic injury or resection of any pathology involving the maxilla. Resultant morbidity depends on the extent of resection. These patients suffer from some cosmetic and functional defects and, in most instances, require rehabilitation. Functional defects can be loss of masticatory efficiency, anosmia, swallowing difficulty, speech deficits, or even loss of vision. Reconstruction of maxillary defects has been a matter of controversy due to the complexity of the native structure. The goals of reconstruction that have been defined include the replacement of the native tissue, creating a seal between the oral cavity and its surrounding structures, restoring structural integrity and mid-face contours, returning normal deglutition and speech, and restoration of masticatory function [1].

Various classification systems have been proposed to quantify the mid-face defect severity. Aramany proposed the initial classification system in 1978, which divided the maxillectomy defects into six different types [2]. The most widely used system was given by Brown et al. in 2000, which classified the defect as vertical and horizontal tissue loss [3]. Another widely used classification system was proposed by Cordeiro and Santamaria which divided the maxillectomy defects into four main types based on the number of walls involved and also proposed their reconstruction algorithm [4]. However, this system did not pay any attention to the need for dental restoration. Okay et al. proposed a comprehensive classification scheme focusing on dentoalveolar restoration, functional outcome, and patient satisfaction [5].

The initial reconstruction options included the obturators with palatal extension depending on the extent of resection. However, these obturators are recommended for a less extensive defect, typically not involving more than 25 percent of the palate [6]. Various local or regional flaps and free tissue transfer have been suggested to reconstruct a complex defect. The local and regional flaps include the palatal island flap, buccal fat pad, and temporalis muscle flap. For vascularized tissue transfer, the options include radial forearm osteocutaneous flap, rectus abdominis soft tissue flap, vascularized iliac crest flap, free fibula flap, or osteocutaneous scapula flap [1, 7].

Virtual surgical planning (VSP) and additive manufacturing have recently gained much impetus in the field of maxillofacial reconstruction. Dental rehabilitation with conventional obturators poses a considerable challenge because of the loss of tissue and bone support which compromises the effects the stability and retention of the prosthesis. This leads to suboptimal prosthodontic rehabilitation. The concept of VSP and rapid prototyping aids the surgeon in virtually planning the surgical resection cuts, designing the reconstruction framework, and simultaneously manufacturing the prototypes as per the design [8]. The fabrication methods are broadly categorized as subtractive and additive methods. Additive manufacturing offers the benefits of reduced manufacturing time and cost, reduced human and production error, and high product density [9]. In this case series, we present four cases of complex maxillary reconstruction, which employed the VSP and additive manufacturing to achieve the desired result (Table 1).

**Table 1:** Characteristic table for patients included in the case series

Scenario no	Gender	Age	Diagnosis	Approach	Defect
1	Male	27	Alveolar defect secondary to trauma	Intra-oral buccal sulcus	Brown’s type Ic
2	Female	55	Pleomorphic adenoma	Intra-oral buccal sulcus	Brown’s type IIb
3	Male	34	Medication related Osteonecrosis of Jaw—Maxilla (MRONJ)	Intra-oral buccal sulcus	Brown’s type IIc
4	Male	19	Juvenile Ossifying fibroma	Intra-oral buccal sulcus	Brown’s type IIIb

## Case Scenario 1 (Fig. 1)

**Fig. 1:**
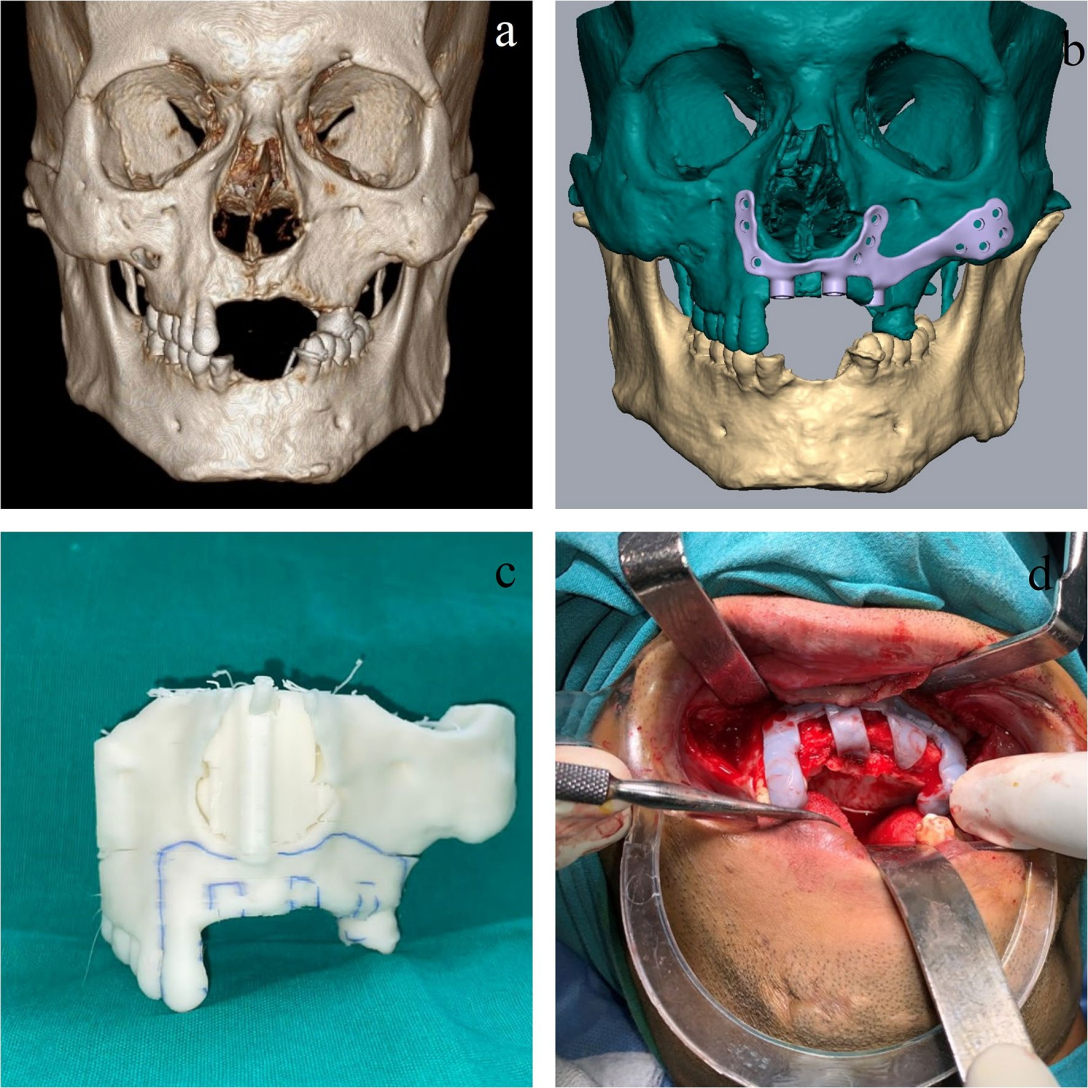
Dental rehabilitation for post-traumatic alveolar defect (Brown Type Ic); **a** Depiction of the edentulous area with respect to 12 to 25 and 33 to 42. Inadequate bone height and width was noted at maxillary alveolus for endosteal implant placement; **b** Framework design by VSP; **c** Marking of osteotomy cuts on stereolithography model for seating of the struts incorporated in the framework; **d** Intra-operative use of surgical guide to accurately place the osteotomy cuts as per the VSP design

A 27-year-old male, who had undergone treatment for fixation of LeFort 1 fracture fixation 3 years ago, wanted dental rehabilitation for the avulsed teeth. The patient had suffered extensive dentoalveolar fracture resulting in avulsion of 12 to 25 and 33 to 42. Patient had been given a removable partial denture following the initial surgery for fracture fixation. However, after 30 months following fixation, patient reported with hardware exposure and also wanted dental rehabilitation with a fixed prosthesis. Repeat radiographic investigations revealed adequate fracture healing. However, the bone width and height were inadequate in the maxillary and edentulous mandibular ridges.

Considering aesthetic concerns and a long bridge span, the patient was planned for a patient-specific subperiosteal implant framework supported fixed prosthesis to rehabilitate the maxillary teeth. However, for the rehabilitation of the mandibular teeth, a decision was taken for a teeth-supported fixed dental prosthesis because of a lack of adequate keratinized gingiva and vestibular depth in the lower arch.

A digital design of the framework was done, which involved the arms extending into the pyriform bilaterally and toward the left zygomaticomaxillary buttress region. Osteotomy design was done on the residual alveolar ridge to house the struts through which the prosthesis would be attached to the framework. A surgical guide was also fabricated, which was used intra-operatively to make the osteotomy cuts accurately. A crestal incision with a bilateral incision was placed, the framework was fixed, and water-tight closure was done. This was followed by acrylic prosthesis fabrication which was attached to the framework through 3 struts. The loading was done 1 month post-surgery.

## Case Scenario 2 (Fig. 2)

**Fig. 2:**
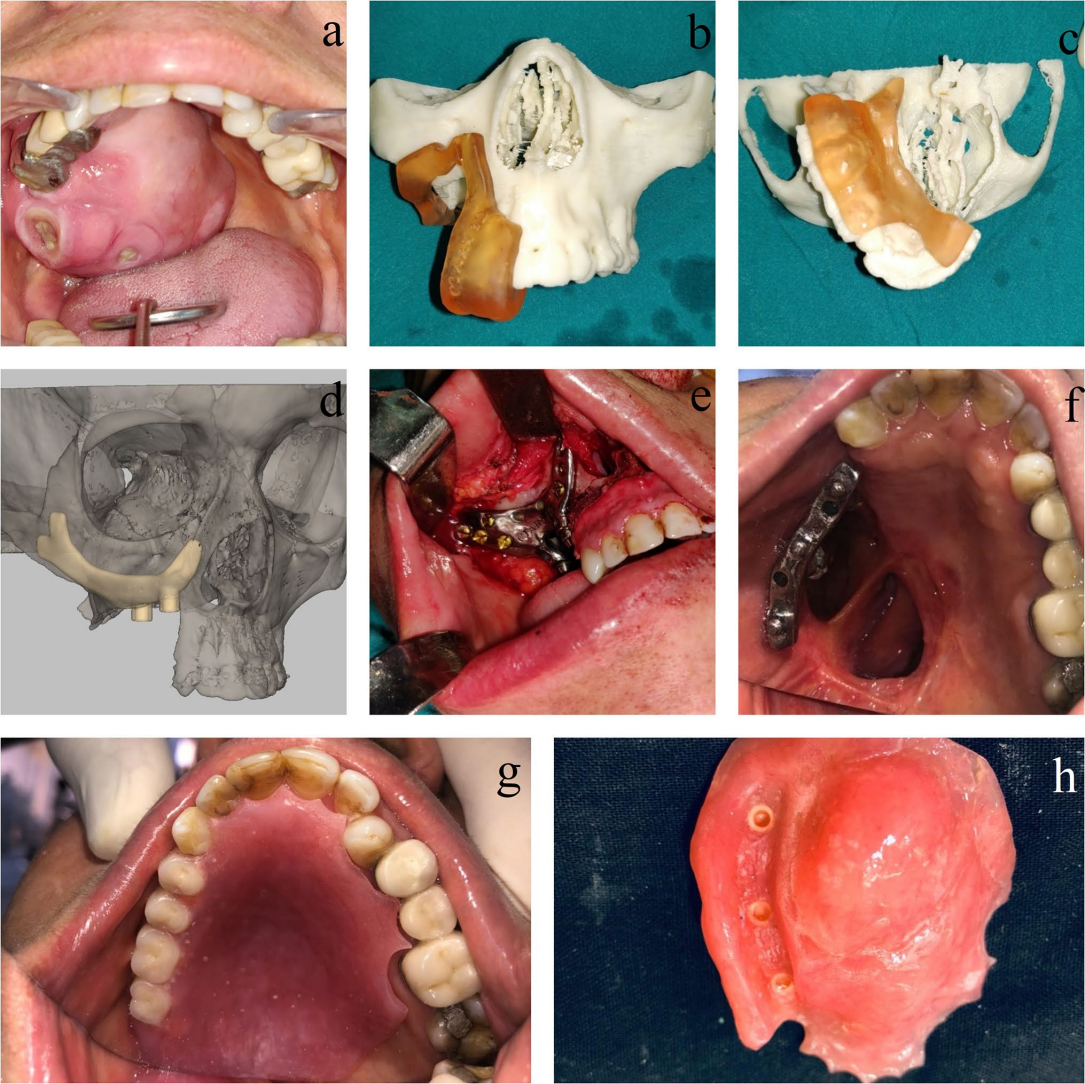
Rehabilitation of maxillary defect due to resection of pleomorphic adenoma (Brown Type IIb); **a** Intra-oral view depicting the tumor involving the right side maxilla and palate; **b** and **c** Use of surgical guide fabricated during VSP to place the accurate osteotomy cuts while tumor resection; **d** VSP showing the design of the framework for rehabilitation of the maxillary defect; **e** Intra-operative fixation of the framework after tumor resection; **f** occlusal view of the bar attachment placed on the struts of the framework after 1 month of healing period (Note the ball attachment on the bar); **g** Occlusal view of prosthesis in place;  **h** View of the prosthesis showing the ball-and-socket attachment

A 55-year-old female reported with swelling of the right side of hard palate. On history, she revealed that she had been diagnosed with pleomorphic adenoma of palate 8 years ago, and had opted for conservative management. However, in the last 2–3 months, the patient noted a rapid increase in the size of swelling with ulceration. A repeat biopsy was done, which confirmed the diagnosis of pleomorphic adenoma.

As the patient was esthetically concerned, she desired immediate reconstruction and functional rehabilitation after tumor resection. Hence, the decision was made to rehabilitate with patient-specific implant-supported prosthesis and palatal obturator.

Virtual surgical planning was done to first mark out the resection margins. A surgical guide was also fabricated to replicate these osteotomy cuts, which was used intra-operatively. A framework design was made on the resected maxilla. The framework had two extensions toward right nasomaxillary buttress and the right zygoma body to dissipate the forces exerted on the framework during functioning. The framework had two struts with ball and socket attachment for the palatal-dento-alveolar prosthesis. The ball and socket kind of attachment was decided as it was easy to remove for the surgeon and aid in disease surveillance during the follow-up. Buccal sulcus incision and palatal incision were carried out to approach for the tumor resection and simultaneous fixation of the framework. Framework fixation to the nasomaxillary buttress and zygomaticomaxillary buttress region was done using 4 (2 × 8 mm) self-tapping screws and 2 (2 × 6 mm) self-tapping screws. Immediately 2-days post-surgery, a customized superstructure of bar-framework was attached to the implant through the two struts. After the initial healing of the surgical site, 3-months post-surgery a dental prosthesis with the palatal obturator component was fixated to this bar attachment by ball and socket attachments.

## Case Scenario 3 (Fig. 3)

**Fig. 3:**
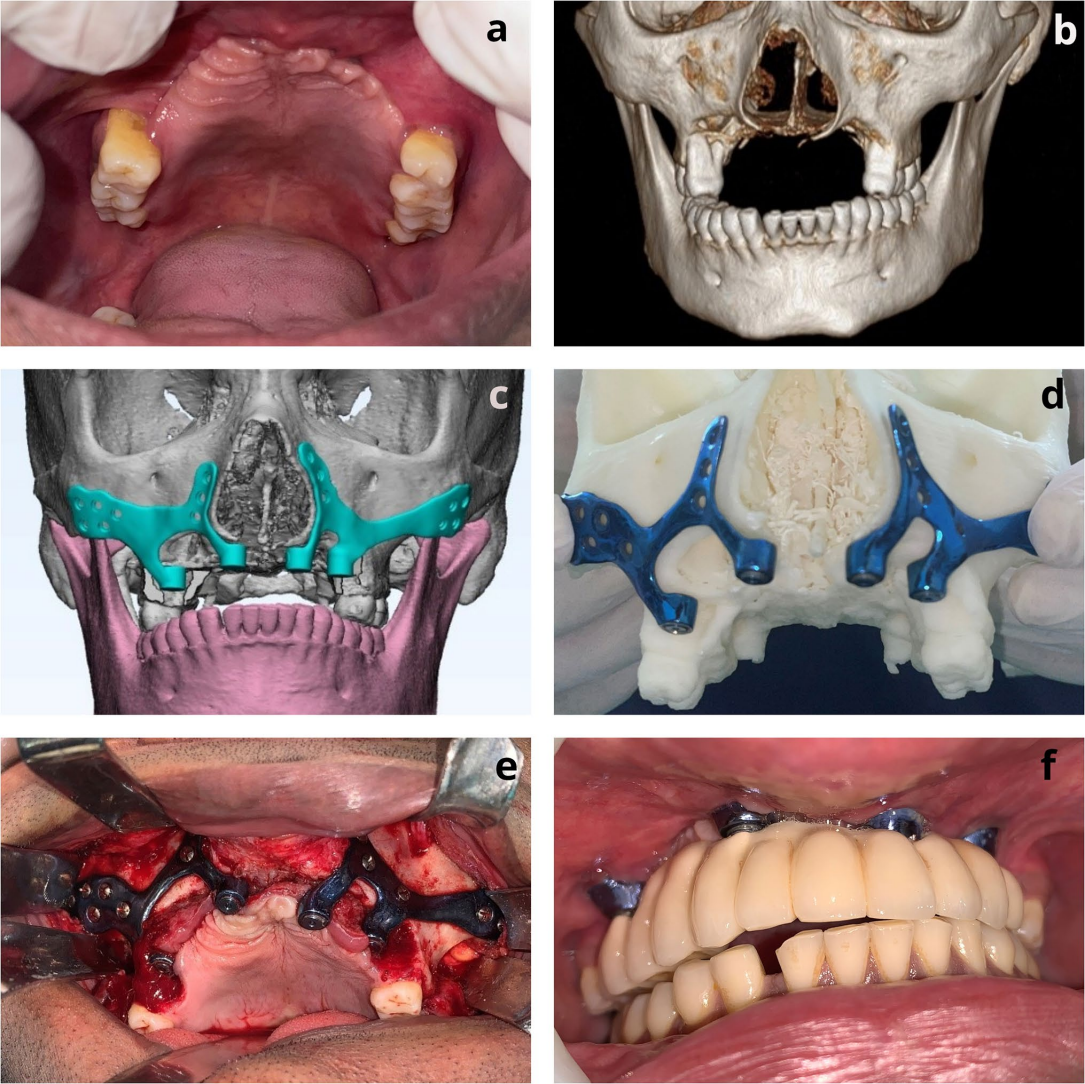
Rehabilitation of MRONJ defect (Brown Type IIc); **a** Intra-oral view of patient’s edentulous anterior maxillary arch resulting due to MRONJ treatment; **b** 3D CT view depicting the paucity of bone in the anterior dentoalveolar region; **c** VSP for designing of framework for rehabilitation of the defect; **d** Fabricated framework seated on the STL model; **e** The framework placement and fixation intra-operatively (depicts the modification of the incision to leave a cuff of crestal tissue to avoid iatrogenic oro-antral communication during surgical exposure).; **f** Intra-oral view of occlusion after fixation of the prosthesis to the framework

A 34-year-old male patient was diagnosed with COVID-19-related medication-related osteonecrosis of the jaw (MRONJ) involving the anterior maxillary segment. Subsequently patient underwent anterior box maxillectomy, under our department, leading to a Brown’s Type IIc defect. The patient also had exposure of the maxillary sinus on the right side which required debridement and primary mucosal closure.

After 9 months of disease-free follow-up, patient was planned for rehabilitation of the defect and the dental prosthesis. Virtual planning was done for framework design. As the defect was extending bilaterally and compromised tissue support in the anterior region, the cross-arch stability with a single framework was deemed inadequate. Hence, two different frameworks on either side of maxilla were designed, which were later interconnected with the bar attachment to give cross-arch stability to balance mediolateral and antero-posterior forces. Each of these frameworks was extended to the pyriform and the zygomaticomaxillary buttress on the respective side of the arch.

Intra-operatively, a modified crestal approach was used, leaving a mucosal tissue in the region of 13, 14 to prevent the oro-antral communication. The right maxillary framework was secured using 4 (1.5 × 6 mm) self-tapping screws in the right zygomaticomaxillary buttress region, 2 (1.5 × 6 mm) self-tapping screws in the right pyriform region. The left maxillary framework was secured using 4 (1.5 × 6 mm) self-tapping screws in the left zygomaticomaxillary buttress region, 2 (1.5 × 6 mm) self-tapping screws in the left pyriform region. After the fixation of the frameworks, a water-tight closure was achieved and a preliminary impression was taken intra-operatively. A bar framework was fabricated, which was secured to the maxillary framework with the four struts.

## Case Scenario 4 (Fig. 4)

**Fig. 4:**
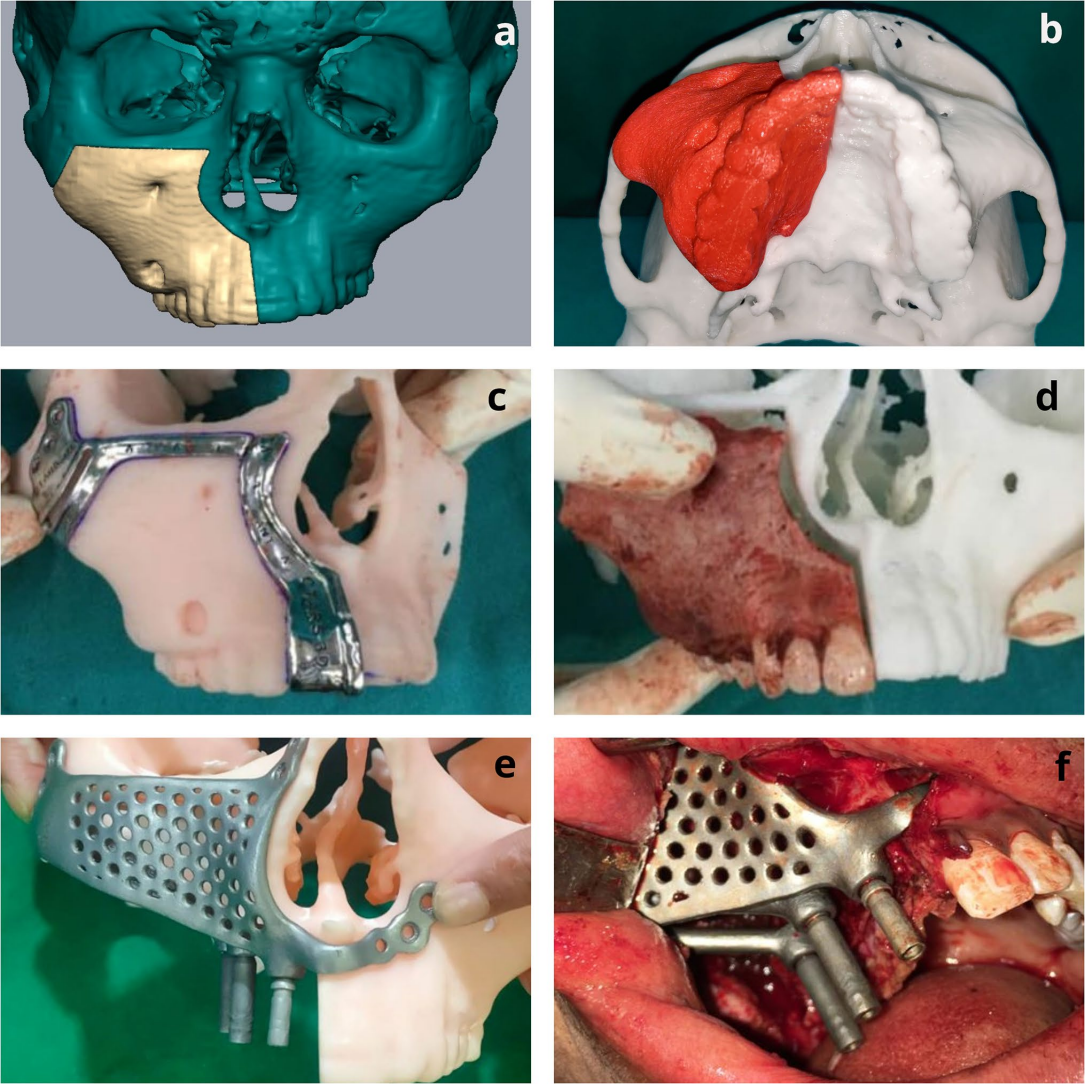
Rehabilitation of maxillary defect resulting due to resection of a benign pathology (Brown Type IIIb); **a** Reconstructed 3D CT cuts from DICOM image depicting the osteotomy cuts placed around the pathology; **b** Fabrication of detachable STL model with cut out resected specimen; **c** Cutting guides placed on the osteotomy models which were used for performing osteotomy intra-operatively; **d** Intra-operative view of the resected specimen, placed alongside the cut out STL model depicting accurate resection as per VSP; **e** Placement of the reconstruction framework on the STL model; **f** Intra-operative view after placement and fixation of the framework

A 19-year-old boy reported with slow-growing swelling of the right-side mid-face region for 1 year. Histopathological examination from the incisional biopsy gave a diagnosis of juvenile ossifying fibroma. CT scan revealed the involvement of the entire right maxilla with inferior-superior extension from the alveolus to the infraorbital foramen. VSP was done to mark out the osteotomy cuts to remove the entire pathology based on the CT images.

Based on this VSP, a stereolithography (STL) model was constructed, which showed the resected specimen separate from the residual maxillary defect. (Fig. 4b–d) This model helped further understand the defect, patient education, and reconstruction planning. An implant framework was designed to facilitate early dental rehabilitation, which took support from the residual zygoma body and the nasomaxillary region. Two strut attachments were incorporated at the alveolus for attachment of the dental prosthesis. Intraoperatively, after completing the osteotomy, the framework was fixed to the right side zygoma region using 4 (1.5 × 8 mm) self-tapping screws, right pyriform region with 2 (1.5 × 6 mm) self-tapping screws and left pyriform region with 2 (1.5 × 6 mm) self-tapping screws. Delayed loading of the prosthesis was done 3 months post-surgery.

## Discussion

Additive manufacturing can be easily integrated with digital imaging technologies to build and design complex physical models and prototype parts. The various technologies used in additive manufacturing are liquid-based processes (ex. Inkjet printing), powder-based printing (ex. 3D printing), and solid-based manufacturing (ex. Laminated object manufacturing) [10, 11]. These technologies can reproduce craniofacial structures and have been used to treat craniomaxillofacial bone defects [12–15]. Stereolithography is the most commonly used additive manufacturing technology application in oral and maxillofacial surgery. It creates medical models for diagnosis, treatment planning, and surgical simulation [16].

Meanwhile, the customized titanium implants and reconstruction plates are manufactured by direct metal laser sintering fabrication technology. In this, the implants are fabricated by applying a focused laser beam to fuse thin layers of powder and metal in a layered manner in a localized region [10, 17]. The 3D VSP, along with additive manufacturing, can be used to plan surgical resections, fabricate the surgical guide, and simultaneously plan and manufacture the implants to reconstruct the ablative defects.

The conventional obturator is the simplest and easiest form of reconstruction in the reconstruction ladder of mid-face defects. Despite being simplistic in approach, these obturators have disadvantages like improper oro-nasal seal leading to regurgitation, inadequate masticatory efficiency, and need for repeated adjustment [6]. On the other hand, reconstruction with flaps or free tissue transfer provides the most physiologic outcomes with respect to speech, deglutition, and formation of an oro-nasal seal. However, they still pose a significant challenge in dental rehabilitation as placement of dental implants in this reconstructed bone requires meticulous planning. The placement of the dental implants is guided by the eventual location of the vascularized bone flap used to reconstruct the alveolus. If this is not planned by VSP, it might lead to placement of dental implants which is not prosthodontically suited [18, 19].

The zygomatic implants have provided an acceptable non-grafting solution for rehabilitation of atrophic maxilla. They can be used in patients with maxillary alveolar defects but with good bone quality in the body of the zygoma region. The long-term functional and esthetic outcomes of zygomatic implant supported rehabilitation has been excellent [20]. However, some of the complications associated with zygomatic implants include risk of orbital injury, speech problems with palatal emergence, post-operative sinusitis, and oro-antral fistula [21]. Further, a lower survival rate of zygomatic implants has been reported in patients with maxillary resection [22]. In our experience, the cost of the patient-specific implants rehabilitation was equivalent to the zygomatic implant supported rehabilitation.

**Fig. 5:**
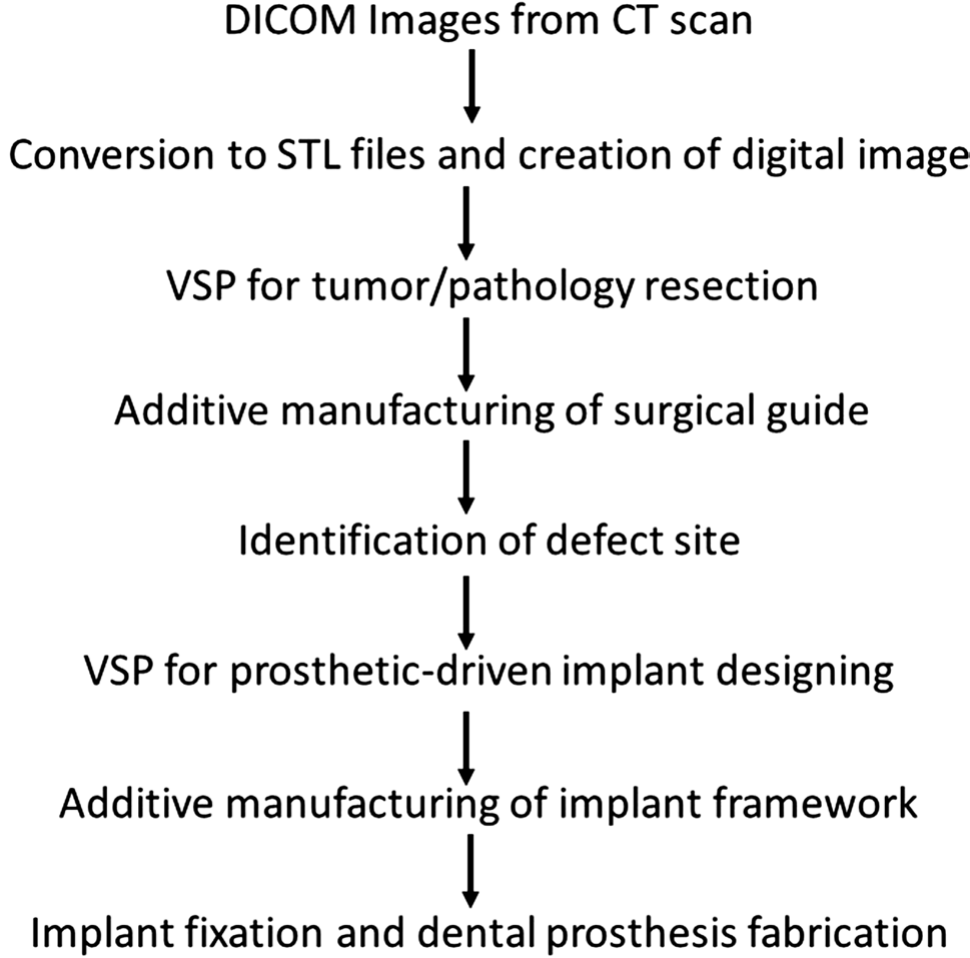
Proposed algorithm for flow of VSP using the prosthetic-driven plan for rehabilitation of maxillary defects by customized implants

In the current case series, the authors used the out-sourced services of the software ‘Geomagic freeform with haptic’ for the VSP. The use of VSP helps the surgeon plan the overall treatment guided by the placement of the final prosthesis. With 3D VSP, the surgeon can precisely mark out the resection margins and replicate it by manufacturing the cutting guides, which is used intra-operatively. After creating this residual defect on the VSP, the surgeon can now plan the prosthetic-driven reconstruction and prevent any compromise in the dental prosthesis. The additive manufacturing technology uses this prosthetic-driven VSP to print the customized framework. (Fig. 5).

This precise guided implant placement carried out during tumor resection offers a huge advantage for patients requiring radiotherapy by hastening the process of dental rehabilitation [23]. Also, owing to the high accuracy of VSP, good control over tumor margins can be achieved by using cutting guides during ablative surgery [24]. Reconstruction of mid-face defect with free-tissue transfer is best suited when extensive soft tissue reconstruction is required. However, customized implant-supported rehabilitation offers significant advantages over free tissue transfer, such as the absence of donor-site morbidity, reduced operating time, and faster recovery for the reconstruction of alveolar defects and low-level maxillectomies. In addition, this prosthesis over customized implants can be made in a semi-fixed manner by incorporating ball-and-socket like attachments, which offers the advantage of disease surveillance.

Recently in the COVID-19 pandemic, there has been an increased incidence of maxillofacial mucormycosis. By June 2021, over 40,000 cases of mucormycosis were reported in India [25, 26]. The standard treatment for these maxillofacial mucormycosis is surgical debridement depending on the extent of infection, leading to various types of maxillectomy defects. This has led to a massive burden of cases requiring rehabilitation of these defects. As with any other maxillectomy defect, the reconstruction option remains the same, which ranges from hollow bulb obturator to free flap reconstruction [27–29]. The option of VSP and additive manufacturing offers a suitable alternative for the rehabilitation of such patients.

Even with all its benefits, VSP can potentially be challenging and have pitfalls of their own [30]. There has to be proper communication between the engineers and the surgeon during the planning, to minimize incorporation of any error. Additionally, the simulation of occlusion in centric relation during the virtual planning can be challenging. For pathology resection planning, the resection margins can vary intra-operatively from the one as determined during the VSP. These modifications intra-operatively can be detrimental to the PSI fixation, if a simultaneous resection and rehabilitation had been planned. It is important to understand that the VSP can work as a useful adjunct and should always be preceded by clinical examination and judgment.

## Conclusion

In conclusion, the integration of two advanced technologies, namely VSP (Virtual Surgical Planning) and additive manufacturing, presents a viable and promising solution for reconstructing complex mid-face or maxillary defects. This approach offers a viable alternative that may not always necessitate extensive soft tissue reconstruction. However, long-term follow-up and controlled trial studies are required to provide an effective comparison with other reconstruction modalities.
